# Consistency of efficacy results across various clinical measures and statistical methods in the lecanemab phase 2 trial of early Alzheimer’s disease

**DOI:** 10.1186/s13195-022-01129-x

**Published:** 2022-12-09

**Authors:** Shobha Dhadda, Michio Kanekiyo, David Li, Chad J. Swanson, Michael Irizarry, Scott Berry, Lynn D. Kramer, Donald A. Berry

**Affiliations:** 1 Eisai Inc, Nutley, NJ USA; 2 Berry Consultants, LLC, Austin, TX USA; 3 University of Texas M.D. Anderson Cancer Center, Houston, TX USA

## Abstract

**Background:**

Lecanemab (BAN2401) is a humanized IgG1 monoclonal antibody that preferentially targets soluble aggregated Aβ species (protofibrils) with activity at insoluble fibrils and slowed clinical decline in an 18-month phase 2 proof-of-concept study (Study 201; ClinicalTrials.gov NCT01767311) in 856 subjects with early Alzheimer’s disease (AD). In this trial, subjects were randomized to five lecanemab dose regimens or placebo. The primary efficacy endpoint was change from baseline in the Alzheimer’s Disease Composite Score (ADCOMS) at 12 months with Bayesian analyses. The key secondary endpoints were ADCOMS at 18 months and Clinical Dementia Rating-Sum-of-Boxes (CDR-SB) and Alzheimer’s Disease Assessment Scale-Cognitive Subscale (ADAS-Cog14) at 18 months. The results have been published previously. Herein, we describe the results of sensitivity analyses evaluating the consistency of the lecanemab efficacy results in Study 201 at the identified dose, the ED90, across multiple statistical methods and multiple endpoints over the duration of the study.

**Methods:**

The protocol-specified analysis model was a mixed model for repeated measures (MMRM). Sensitivity analyses address the consistency of the conclusions using multiple statistical methods. These include a disease progression model (DPM), a natural cubic spline (NCS) model, a quadratic mixed model (QMM), and 2 MMRMs with additional covariates.

**Results:**

The sensitivity analyses showed positive lecanemab treatment effects for all endpoints and all statistical models considered. The protocol-specified ADCOMS analysis showed a 29.7% slower decline than placebo for ADCOMS at 18 months. The various other analyses of 3 key endpoints showed declines ranging from 26.5 to 55.9%. The results at 12 months are also consistent with those at 18 months.

**Conclusions:**

The conclusion of the primary analysis of the lecanemab Study 201 is strengthened by the consistently positive conclusions across multiple statistical models, across efficacy endpoints, and over time, despite missing data. The 18-month data from this trial was utilized in the design of the confirmatory phase 3 trial (Clarity AD) and allowed for proper powering for multiple, robust outcomes.

## Introduction

Lecanemab (BAN2401) is a humanized IgG1 monoclonal antibody that preferentially targets soluble aggregated Aβ (protofibrils) with activity at insoluble fibrils [1–6]. In animal models, the reduction of Aβ protofibrils and Aβ plaque, as well as prevention of Aβ deposition before plaques develop, has been demonstrated using the murine version of lecanemab [1, 2]. Lecanemab has been evaluated in two phase 1 studies (study BAN2401-A001-101 [101] in subjects with mild to moderate Alzheimer’s disease (AD) and study BAN2401-J081-104 [104] in Japanese population with early AD) [7].

Study 201 was an 18-month phase 2 proof-of-concept, multicenter, double-blind, placebo-controlled, phase 2 dose-finding study (Study 201) conducted in 856 subjects with early AD [8]. Response-adaptive randomization was implemented following a fixed randomization of 196 subjects among five lecanemab dose regimens: 2.5 mg/kg bi-weekly, 5 mg/kg monthly, 5 mg/kg bi-weekly, 10 mg/kg monthly (10M), and 10 mg/kg bi-weekly (10BW), or placebo. The primary goal of the study was to determine the ED90, the simplest dose that achieves ≥ 90% of the maximum treatment effect, on the Alzheimer’s Disease Composite Score (ADCOMS) at 12 months of treatment, based on a Bayesian dose-response model. Analyses of the 12-month clinical change on ADCOMS were performed per a prespecified schedule to determine future patient allocation if futility or an early success decision was not achieved.

To have greater confidence in the efficacy of the drug should early success be declared, the early success criterion had a high hurdle, namely, super-superiority over placebo which required a 95% probability of greater than a 25% clinical reduction in decline versus placebo. If an early success declaration was made, enrollment would stop and all subjects in the trial would be followed through 18 months. The sponsor would be notified that super-superiority had been met, and this would accelerate initiating a phase 3 trial. If the study did not stop for futility or super-superiority, then the final analyses of clinical change on ADCOMS at 12 months and at 18 months would be performed with a threshold of 80% probability for the declaration of super-superiority. Dose 10BW was identified as the ED90, which had 64% and 76% probabilities of super-superiority over placebo at 12 and 18 months, respectively. The probabilities of superiority to placebo are 97.5% and 97.7% at 12 months and 18 months, respectively. Super-superiority required at least a 25% improvement relative to placebo; superiority did not have a specific improvement requirement.

Other key efficacy endpoints included Clinical Dementia Rating-Sum-of-Boxes (CDR-SB) and Alzheimer’s Disease Assessment Scale-Cognitive Subscale (ADAS-Cog14) all at 18 months.

The protocol-specified analysis method for each key endpoint was a mixed model for repeated measures (MMRM). At 18 months of treatment, there was less decline in all 3 clinical endpoints for the 10BW dose compared to placebo for ADCOMS (two-sided *p* = 0.034, percentage slowing = 30%), ADAS-Cog14 (two-sided *p* = 0.017, percentage slowing = 47%), and CDR-SB (two-sided *p* = 0.125, percentage slowing = 26%).

Herein, we describe the results of the sensitivity analyses evaluating the consistency of the lecanemab efficacy results in Study 201 at the chosen dose, the ED90, across multiple statistical methods and multiple endpoints as well as over follow-up time.

## Methods

In Study 201, a total of 856 subjects were randomized, and 854 were treated (lecanemab 609; placebo 245) between December 2012 and November 2017 at 117 sites. The final allocation of subjects to the treatment arms via adaptive randomization is shown in Table 1. The Bayesian model in this study identified the 10BW arm as the most likely ED90.

**Table 1:** Allocation of subjects based on response adaptive randomization and available for analysis

	Placebo	2.5 mg/kg bi-weekly (2.5BW)	5 mg/kg monthly (5M)	5 mg/kg bi-weekly (5BW)	10 mg/kg monthly (10M)	10 mg/kg bi-weekly (10BW)	Total
**Number of subjects randomized**	247	52	51	92	253	161	856
					414		
**Number of subjects in efficacy analyses**	238	52	48	89	246	152	825
					398		

Secondary endpoints of change from baseline in CDR-SB and ADAS-Cog14 at 18 months for Study 201 have been analyzed with Bayesian and conventional approaches [8]. To assess the robustness of efficacy results, we conducted sensitivity analyses for all 3 key secondary clinical endpoints using different statistical methods. All analyses were conducted based on a modified intention-to-treat population, which included data from participants who had baseline and at least one post-baseline efficacy measurement. All analyses (except the protocol-specified one) were post hoc, and all pairwise tests of treatment effects were conducted at a two-sided alpha level of 0.05 without multiplicity adjustment. The point estimate of treatment effect is provided at each post-baseline visit including 12 months and 18 months. The analyses include all lecanemab doses (except the DPM model as stated below), but only placebo, lecanemab 10M, and lecanemab 10BW are presented, since the sample size from other treatment groups was small, especially relative to the model parameters to be estimated.

In addition to the MMRM analysis model specified in the protocol (pMMRM), we considered the following 5 statistical analysis models: disease progression model (DPM), natural cubic spline model (NCS), quadratic mixed model (QMM), and 2 additional MMRMs with extra terms included (aMMRM1 and aMMRM2). In all 6 models, the outcome is the change from the baseline of the respective clinical endpoint at each scheduled post-baseline time point. The common model terms include treatment, baseline value, randomization stratification variables, and geographical region. The model terms for each model are specified in Table 2.

**Table 2:** Model terms included in 6 analysis models

Model	Model terms	Note
pMMRM	Treatment, baseline value, randomization stratification variables^a^, geographical region, visit, treatment-by-visit interaction	This model is defined in the study SAP. Visit is a discrete model term.
aMMRM1	Treatment, baseline value, randomization stratification variables, geographical region, visit, treatment-by-visit interaction, baseline-by-visit interaction	Compared to pMMRM, this model has one additional term: baseline-by-visit interaction. Including baseline-by-visit interaction in the MMRM analysis has been the current practice [9, 10].
DPM	Treatment, baseline value, randomization stratification variables, geographical region, week	Week is included as a discrete term. This model assumes the DPR; the rate of the decline of the treatment group to the rate of decline of the placebo group is constant across study weeks. The model is valid and likely more powerful than MMRM if the assumption is true.
NCS	Treatment, baseline value, randomization stratification variables, geographical region, treatment-by-visit interaction, natural cubic spline (NCS), treatment-by-NCS interaction, baseline-by-NCS interaction	Similar to aMMRM but with visit replaced by a natural cubic spline with 2 degrees of freedom (as a continuous function of week). This model assumes non-linear non-parametric disease progression over time.
QMM	Treatment, baseline value, randomization stratification variables, geographical region, week, treatment-by-week interaction, baseline-by-week interaction, week^a^week (week2), treatment-by-week2 interaction, baseline-by-week2 interaction	Similar to aMMRM terms but “week” in place of visit with week being included as a continuous term. This model assumes quadratic disease progression over time.
aMMRM2	Treatment, baseline value, randomization stratification variables, geographical region, visit, treatment-by-visit interaction, baseline-by-visit interaction, ApoE4 -by-visit interaction, ApoE4-by-treatment interaction, ApoE4-by-treatment-by-visit interaction	Compared to aMMRM1, this model has all 2-way interaction terms and 3-way interaction terms among treatment, ApoE4 status, and visit. This model is good to explore the impact of ApoE4 on treatment comparison when ApoE4 is not balanced between the comparison groups.

^a^Randomization stratification variables include: clinical subgroup (MCI due to AD vs mild AD), use of ongoing AD treatment at baseline (yes vs no), and ApoE4 status (positive vs negative)

Compared to pMMRM, aMMRM1 has an additional term: baseline-by-visit interaction. The inclusion of an interaction term of baseline by visit is consistent with other analysis models [9, 10]. To evaluate the impact of ApoE4 status on disease progression, the aMMRM2 was utilized, which includes all interaction terms among ApoE4 status, treatment, and visit. NCS is a model similar to aMMRM but with visit replaced by a natural cubic spline with 2 degrees of freedom [11]. The QMM model is the same as aMMRM, but it utilizes a continuous time and a quadratic time effect.

DPM is a disease progression model assuming a proportional treatment effect over time [12]. This model evaluated the disease progression ratio (DPR), which is defined as the rate of decline of the treatment group to the rate of decline of the placebo group. Only the placebo, 10M, and 10BW treatment groups were included in this DPM model.

## Results

### Sensitivity analyses results

**Fig. 1:**
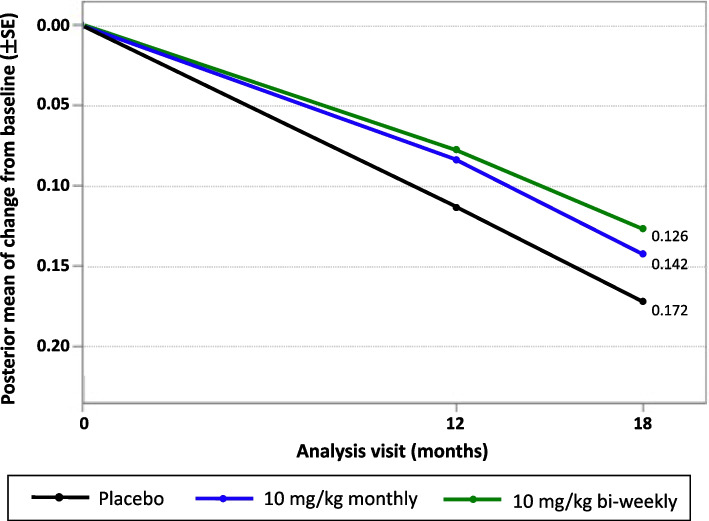
Bayesian posterior mean change in ADCOMS from baseline over time

The Bayesian posterior mean change in ADCOMS from baseline at 12 and 18 months for all doses, including 10BW and 10B, are shown in Fig. 1. At 12 months, the mean difference from placebo for 10BW is − 0.037, with a 97.5% probability of superiority to placebo. The 18-month mean difference from placebo for 10BW is − 0.047 (− 0.093, − 0.001), with a 97.7% probability of superiority to placebo.

**Fig. 2:**
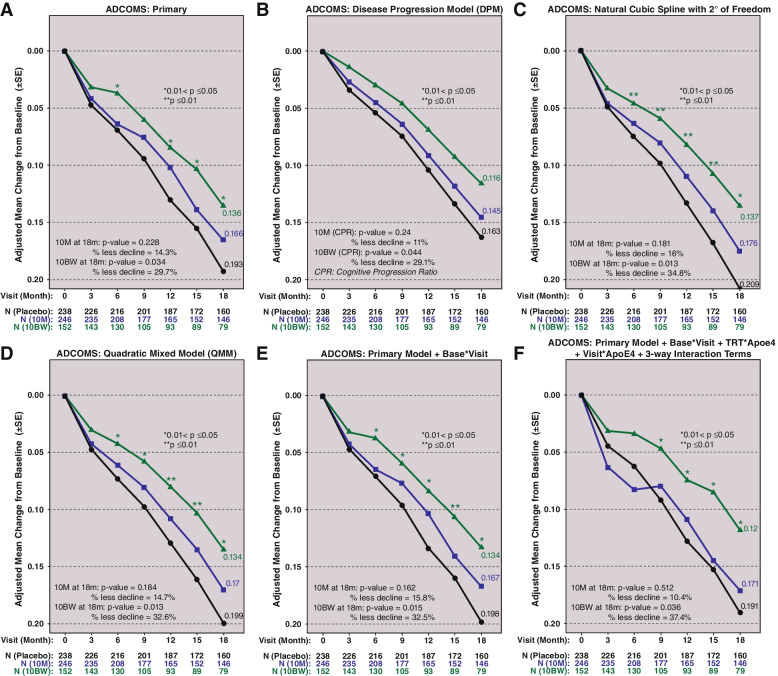
ADCOMS results for dose regimens 10M and 10BW versus placebo across the statistical methods. **A** Primary. **B** DPM. **C** Natural cubic spline. **D** QMM. **E** Primary model + base * visit. **F** Primary model + base * visit + TRT * Apoe4 + visit * Apoe4 + 3-way interaction terms. *P* values are two-sided

Sensitivity analyses estimate that 10BW and 10B slowed the rate of clinical disease for ADCOMS, CDR-SB, and ADAS-Cog14 at 18 months across all sensitivity statistical models. The model estimates for 18-month ADCOMS are shown in Fig. 2. Panel A shows the pre-specified pMMRM analysis, with an estimated slowing of decline for 10BW versus PBO of 29.7%, with a steady and consistent widening of the curves over time, and with a statistically significant separation from 12 through 18 months. Panels B through F include the results from additional analyses. The results across different models are consistent with the pMMRM analysis results: For 10BW, the estimated slowing at 18 months ranges from 29.1 to 37.4% with statistically significant differences (two-sided *p* < 0.05) from 9 months to 18 months and with separation from placebo starting as early as at 6 months for 4 additional analysis models. The DPM model estimated the disease progression in 10BW to be significantly slower compared to placebo during the study (*p* = 0.044).

**Fig. 3:**
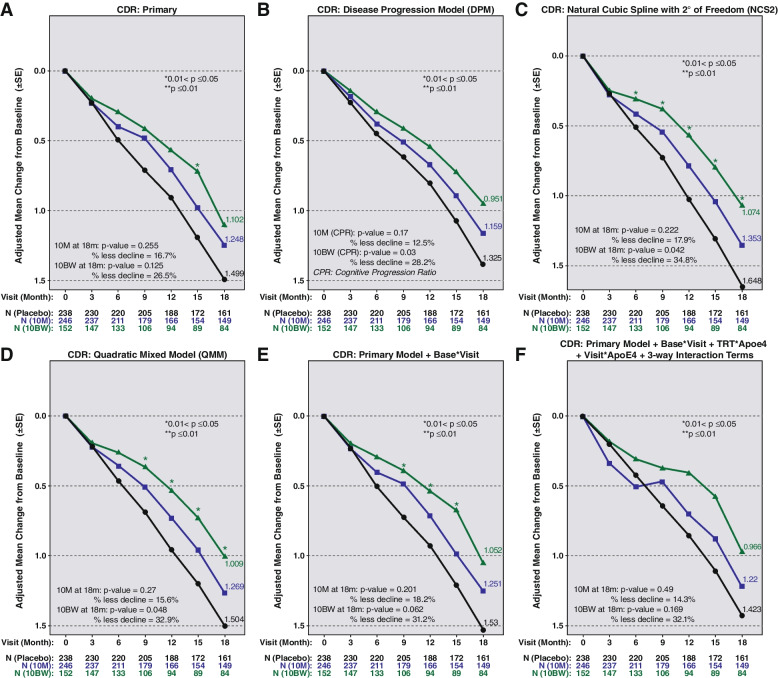
CDR-SB results for dose regimens 10M and 10BW versus placebo across the statistical methods. **A** Primary. **B** DPM. **C** Natural cubic spline. **D** QMM. **E** Primary model + base visit. **F** Primary model + base * visit + TRT * Apoe4 + visit * Apoe4 + 3-way interaction terms. *P* values are two-sided

The results of the multiple analyses for CDR-SB are depicted in Fig. 3. Panel A shows the pre-specified pMMRM analysis, with estimated slowing for 10BW versus PBO of 26.5%, again with the steady and consistent widening of the curves over time. Panels B through F show the percent slowing ranging from 28.2 to 38.4% across the different statistical models.

**Fig. 4:**
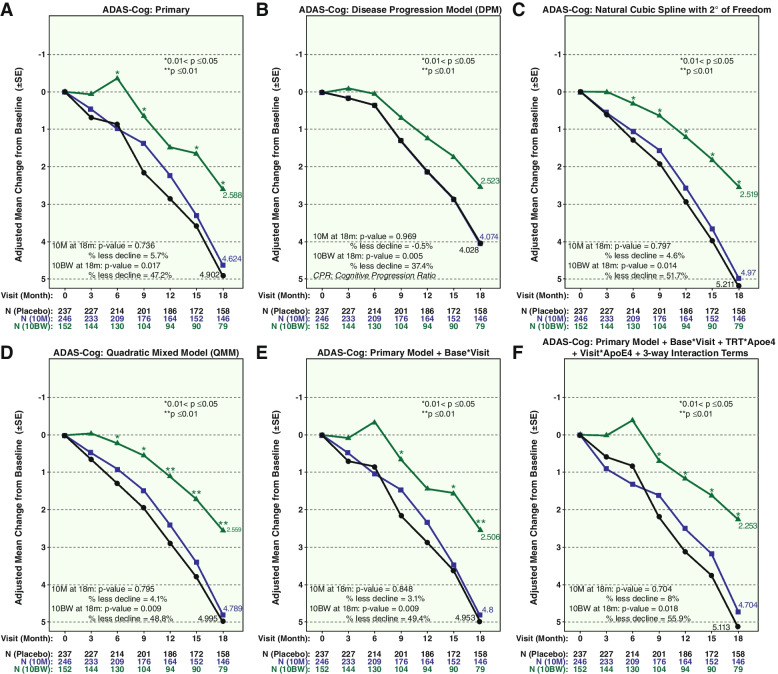
ADAS-Cog14 results for dose regimens 10M and 10BW versus placebo across the statistical methods. **A** Primary. **B** DPM. **C** Natural cubic spline. **D** QMM. **E** Primary model + base visit. **F** Primary model + base * visit + TRT * Apoe4 + visit * Apoe4 + 3-way interaction terms. *P* values are two-sided

The results of the multiple analyses for ADAS-Cog14 are shown in Fig. 4. The pre-specified pMMRM analysis in panel A shows an estimated slowing for 10BW versus PBO of 47.2%, again with the steady and consistent widening of the curves over time albeit with somewhat greater variability than the other two measures. Panels B through F show the estimated slowing ranging from 37.4 to 55.9% across different statistical models.

## Discussion

Lecanemab has demonstrated a reduction in brain amyloid accompanied by a consistent slowing of clinical decline across several clinical endpoints in Study 201. This study supported the therapeutic concept of targeting specific soluble aggregate species (protofibrils) in the process of pathophysiological amyloid generation in AD. The study identified 10BW as the ED90, the smallest lecanemab dose that achieved ≥ 90% of the maximum treatment effect among doses considered. At 12 months, 10BW had a 63.8% probability of super-superiority to placebo, less than the targeted 80%, and a 97.5% probability of superiority to placebo, analogous to a one-sided *p*-value of 0.025. At 18 months, 10BW had a 76.0% probability of super-superiority to placebo and a 97.7% probability of superiority to placebo, analogous to a one-sided *p*-value of 0.023 [8].

In our study, we addressed the sensitivity of the efficacy conclusions of lecanemab 201 based on the statistical methods used. We found that the conclusions of the primary analysis method are quite robust with respect to variations in statistical methodology. We also observed robustness in the choice of clinical endpoint. Overall, these analyses conclude the consistency of efficacy results across different statistical models with 3 clinical measures, ADCOMS, CDR-SB, and ADAS-Cog14. Our results show that our sensitivity analyses draw very similar conclusions to those of the pre-specified MMRM analyses and the primary Bayesian analysis.

The US Food and Drug Administration has promoted innovation in clinical trial design via the Bayesian approach; does that make clinical trials more efficient? We have found that the Bayesian design of lecanemab trial 201 assigned most subjects to better-performing doses. It is found in the context of missing data that the most effective dose of lecanemab nearly doubles its estimated efficacy at 18 months in comparison with restricting to subjects who completed 18 months.

The consistency of these sensitivity analyses helps confirm the usefulness of the Bayesian design of lecanemab Study 201. The Bayesian approach can improve the efficiency of drug development and the accuracy of clinical trials, even in the context of substantial data missingness. For example, lecanemab Study 201’s Bayesian design utilized multiple imputation [13], which was useful in accommodating unanticipated missing data due to regulatory authority-mandated dose-specific drop-outs [8]. Innovations associated with using the Bayesian approach improve the efficiency of drug development and the accuracy of clinical trials, even when there is substantial data missingness.

### Limitations

Our study has some limitations. Most of these analyses are retrospective and so should be considered as descriptive in nature. The *p*-values we give are nominal and do not consider the multiplicities of endpoints, types of analyses, and timing of analyses. There are many possible statistical analyses of any dataset of which we have provided a relatively small number. We chose commonly proposed candidates for alternative analyses as being representative of the gamut of possibilities. We followed through on and provide here the results of all the analyses we initially considered. In particular, we did not reject any analyses after seeing their results.

### Summary/conclusion

We demonstrate that the clinical efficacy results in the lecanemab phase 2 Study 201 are consistent across endpoints, statistical methodology, and over time. In particular, the Bayesian and conventional analyses of ADCOMS results at 18 months are supported by robust conclusions for CDR-SB and ADAS-Cog14 across five other statistical methods. Two phase 3 studies have been initiated with the goal of confirming lecanemab’s efficacy and safety (Clarity AD [NCT03887455] in early AD and AHEAD [NCT04468659] in preclinical AD). The phase 2 Study 201 18-month data were utilized and allowed for properly powering the Clarity AD trial, which recently reported positive preliminary results [14], for multiple, robust outcomes.

## Study highlights

Lecanemab, a humanized IgG1 monoclonal antibody that preferentially targets soluble aggregated Aβ species (protofibrils), was evaluated in an 18-month, multicenter, double-blind, placebo-controlled, phase 2 proof-of-concept and dose-finding study (Study 201; ClinicalTrials.gov NCT01767311) in 856 subjects with early Alzheimer’s disease (AD). However, the results of sensitivity analyses evaluating the consistency of the lecanemab efficacy results in Study 201 at the identified dose across multiple statistical methods and multiple endpoints over the duration of the study have yet to be published.

We show that the primary analysis of the lecanemab Study 201 is strengthened by the consistently positive conclusions across multiple statistical models, across efficacy endpoints, and over time, despite missing data. The 18-month data from this trial was utilized in the design of the confirmatory phase 3 trial (Clarity AD) and allowed for proper powering for multiple, robust outcomes.

## Data Availability

The datasets generated during and/or analyzed during the current study are available from the corresponding author upon reasonable request.
